# The association between analgesic drug use in pregnancy and neurodevelopmental disorders: protocol for an umbrella review

**DOI:** 10.1186/s13643-020-01465-9

**Published:** 2020-09-02

**Authors:** Janell Kwok, Hildigunnur Anna Hall, Aja Louise Murray, Bonnie Auyeung

**Affiliations:** 1 Department of Psychology, University of Edinburgh, 7 George Square, Edinburgh, EH8 9JZ UK; 2 Department of Psychiatry, Autism Research Centre, University of Cambridge, Douglas House, 18b Trumpington Road, Cambridge, CB2 8AH UK

**Keywords:** Umbrella review, Analgesics, Medication, Painkillers, Pregnancy, Maternal, Autism, ADHD

## Abstract

**Background:**

Maternal prenatal health has been shown to be an important influence on children’s developmental outcomes, which has led to an increased emphasis on providing more information to support clinical decisions in pregnancy. Several systematic reviews suggest that analgesic drug use during pregnancy may have neurodisruptive properties. However, no firm conclusions have yet been drawn on the associations between prenatal analgesic drug use and children’s long-term development of neurodevelopmental disorders such as autism spectrum disorder (ASD) or attention deficit hyperactivity disorder (ADHD). Therefore, an umbrella review is proposed for the purpose of examining the associations between maternal analgesic drug use during pregnancy and diagnoses of neurodevelopmental disorders.

**Methods:**

Included systematic reviews will consist of studies examining the effect of maternal prenatal analgesic drug use, specifically ibuprofen, acetaminophen, aspirin, naproxen, diclofenac, and ketoprofen, on children’s neurodevelopmental disorder status. Examined drugs were restricted to those readily accessible and frequently used by pregnant women, and with characteristics that allow them to cross the placenta and directly affect fetal development. Outcomes will be restricted to formal clinical diagnoses of ASD and/or ADHD. Two reviewers will independently identify eligible reviews from six databases (e.g., PubMed, EMBASE, PsychINFO) from inception dates of databases to the date of data extraction, and conduct manual searches of reference lists, consultation with field experts, and scan of pre-print archives. Extracted data will also include short qualitative summaries by both reviewers. As part of quality assessment, a standardized measurement tool to assess systematic reviews (AMSTAR 2) will be used. A narrative synthesis is proposed to integrate findings from different, potentially methodologically heterogeneous, studies.

**Discussion:**

This umbrella review of associations between maternal prenatal use of analgesic drugs and children’s neurodevelopmental disorders could allow for firmer conclusions to be drawn through the synthesis of all relevant published research. The synthesis of findings using high-quality evidence could provide more accurate healthcare information on the long-term effects of analgesic drugs on neurodevelopment, to better guide future clinical decisions during pregnancy. This review will also allow gaps and methodological differences in the literature to be identified, informing recommendations for future research.

**Systematic review registration:**

PROSPERO CRD42020179216.

## Background

Recent years have shown an increased emphasis on the effects of maternal prenatal health on children’s long-term development. Decisions made during pregnancy should be as well-informed as possible to understand possible long-term effects, such as on children’s neurodevelopment. Since 1990, there has been global increased awareness of neurodevelopmental disorders such as autism spectrum disorder (ASD) and attention deficit hyperactivity disorder (ADHD), with the worldwide prevalence of ASD being 0.17–0.62% [1] and 5.29–7.20% for ADHD [2, 3]. Currently, 56% of pregnant women are reported to use analgesic drugs [4]. With this prevalence rate, it is worthwhile to examine the effects of prenatal drug use on a child’s long-term development.

With increased cardiac output and blood flow that occurs as part of the physiological changes during pregnancy, drug absorption also increases [5]. This bioavailability creates complex chemical changes in both the mother and fetus. Studies on pharmacokinetic changes in pregnancy show how placenta transfer occurs between maternal and fetal blood circulatory systems [6]. Some pharmacological characteristics of drugs that cross the placenta include soluble lipids, unbound drugs that are lower of degree of ionization and have a molecular weight of less than 500 g/mol [7]. Drugs that fall under this category include aspirin, ibuprofen, naproxen, and acetaminophen (paracetamol). With hormonal changes in pregnancy and increased lipid levels diminishing the binding capacity of drugs [8], a fetus may experience large concentrations of drug doses, directly affecting the developing brain.

Some medications of interest in this review, such as acetaminophen and naproxen, are also antipyretic drugs. Antipyretic drugs are prostaglandin antagonists which affect the hypothalamus and decrease body temperatures during an episode of fever. Untreated fever in pregnancy has been associated with malformations in children, particularly neural tube defects, heart defects, and oral clefts [9]. While there is some evidence that antipyretic medication has a protective effect, some pregnant women may use these medications without considering potential long-term effects on their child’s development neurodevelopment [9]. As some women avoid mild analgesics/antipyretics during pregnancy [10], women therefore need to carefully weigh the risks of untreated fever against the risk of using mild analgesics/antipyretics.

During pregnancy, drugs may be used for a wide variety of reasons, such as to alleviate pain, improve health, or increase well-being. While previous research has suggested neurodisruptive properties of certain drugs on the fetus [11], some of these drugs are still not recognized as human teratogens and are readily accessible to the public. Current studies show that prenatal use of drugs is frequent in pregnant women, with around 90% of them taking some form of medication during pregnancy [12]. With analgesic drugs recorded as the most commonly recommended class of drugs to be used during pregnancy, it is possible that pregnant women are engaging in this type of drug use without being aware of potential long-term effects on their child.

Recognizing that there was insufficient information to guide clinical decisions, the US Food and Drug Administration (FDA) established new pregnancy exposure registries in 2002 in order to encourage the use of prospective studies to obtain relevant data [13]. This registry is similar to those in other countries, such as the Swedish Medical Birth Register, which was established in 1973, and has collected information on drugs used during pregnancy since 1995 [14]. However, at the time of writing, neither of these registers have produced research with firm conclusions regarding prenatal exposure to analgesic drugs and their influence on children’s neurodevelopmental outcomes.

Since January 2016, the FDA also proposed changing medication labels in order to provide more information for pregnant women. Despite having shown that analgesic drugs cross the placenta, most of them have been placed under either FDA categories:

category B:

“Animal reproduction studies have failed to demonstrate a risk to the fetus and there are no adequate and well-controlled studies in pregnant women, or animal studies have shown adverse effects, but adequate and well-controlled studies in pregnant women have failed to demonstrate a risk to the fetus in any trimester,”

or category C:

“Animal reproduction studies have shown an adverse effect on the fetus and there are no adequate and well-controlled studies in humans, but potential benefits may warrant use of the drug in pregnant women despite potential risks,”

or category D:

“There is positive evidence of human fetal risk, but the benefits from use in pregnant women may be acceptable despite the risk (e.g., if the drug is needed in a life-threatening situation or for a serious disease in which safer drugs cannot be used or are ineffective)” [15, 16].

This illustrates that current clinical guidelines are still based on limited and inconsistent evidence regarding the long-term effects of these drugs on the fetus.

Research has suggested that prenatal drug use is associated with increased behavioral symptoms such as conduct problems and hyperactivity at age 7 [17]. Further, a systematic review of nine studies suggested that prenatal exposure to analgesic drugs such as acetaminophen was associated with an increased risk of neurodevelopmental disorders between 18 months and 3 years [18]. These results are consistent with another systematic review of seven retrospective cohort studies [11] that found a significantly increased risk of ASD and ADHD in children with age ranges of 3 to 12 years old, in relation to prenatal acetaminophen use. However, other reviews question conclusions on links between prenatal analgesic drug use and neurodevelopmental disorders in children and suggest that findings may be influenced by unmeasured confounding [19, 20]. The above studies and national registries imply that current research or government initiatives are not yet sufficient in nature to understand long-term effects of prenatal analgesic drug use on a child’s development. Thus, an umbrella review is needed to provide a comprehensive overview of the existing evidence in this field.

Systematic reviews have long been held as the gold-standard in contributing to evidence-based healthcare by informing decision-making processes [21]. The next step in conducting an umbrella review offers valuable insight through providing an overall summary of multiple systematic reviews; effectively synthesizing, comparing, and contrasting results of published systematic reviews and meta-analyses. Umbrella reviews are conducted in line with the same principles as systematic reviews, in terms of, for example, predefined search strategies, quality assessment, and reporting guidelines. However, the subject of an umbrella review is existing systematic reviews and meta-analyses, rather than original research studies [21, 22]. The objectives the proposed umbrella review also align with Cochrane’s overview of reviews, consisting of (1) a clearly formulated objective to answer a specific research question, (2) inclusion of only systematic reviews, (3) explicit and reproducible methods of identification and risk/quality assessment, (4) collection and presentation of data from studies (description of systematic reviews, risk of bias, quantitative outcome data, certainty of evidence using a clinical outcome framework such as GRADE assessments), and (5) discussion of findings related to specific research questions (i.e., summary of main results, completeness, applicability and quality of evidence, agreements, and/or disagreements with other studies) [23]. The topic of associations between maternal prenatal use of analgesic drugs and children’s neurodevelopmental disorders has reached a level of maturity where it can benefit from this form of synthesis, further allowing for firmer conclusions to be drawn through an overall examination of published research.

The aims of the proposed umbrella review are to (a) summarize and synthesize findings from systematic reviews or meta-analyses on links between analgesic drug use in pregnancy and children’s diagnoses of neurodevelopmental disorders, specifically ASD and ADHD; (b) use high-quality evidence to provide firm conclusions from current literature, in order to inform healthcare guidance for pregnant women; and (c) identify gaps and methodological weaknesses in the literature to inform recommendations for future research in this area.

## Methods

### Registration and reporting information

The umbrella review protocol is being reported in accordance with the reporting guidance provided in the Preferred Reporting Items for Systematic Reviews and Meta-Analyses Protocols (PRISMA-P) statement [24] (Additional File 5). This protocol has been registered within the International Prospective Register of Systematic Reviews (PROSPERO) database (registration ID: CRD42040179216).

### Inclusion criteria

Included studies will be based on the following eligibility criteria:

#### Population

Study populations will include pregnant women and the children resulting from their pregnancies. No age limit is set for the pregnant women nor their offspring.

#### Exposures

Only systematic reviews or meta-analyses examining the effects of analgesic drugs will be included in this review. Drugs reviewed will fulfill the criteria of easy access and common usage [25] and have characteristics that allow them to cross the placenta and directly affect the development of the fetus [6]. This umbrella review will focus on reviews of the following specific medications: ibuprofen, acetaminophen, aspirin, naproxen, diclofenac, and ketoprofen. ASD and ADHD assessed by any means (e.g., clinical diagnoses, parent- or teacher-report) [11] will be included in this review. All types of autism spectrum disorders (e.g., pervasive developmental disorder (not otherwise specified) and Asperger’s disorder) will be included in this review. All types of attention deficit hyperactivity disorders (e.g., attention deficit disorders, hyperkinetic disorders) will be included in this review.

#### Setting

Healthcare settings will include both hospital and community data. The study will cover the analgesic drugs taken during the period of pregnancy only.

#### Outcomes

All reviews which draw on indications of ASD, ADHD, or co-occurring ASD and ADHD, assessed by any means, as outcomes will be included, as will gender-specific reviews. No upper age limit restrictions will be applied for study outcomes.

#### Study design

Only reviews of studies which include human offspring will be considered for this review. Only reviews of quantitative studies will be included. The review will be restricted to systematic reviews and meta-analyses but will not be restricted to reviews of studies of a particular design (e.g., longitudinal or cross-sectional). Methodological differences will be discussed in the umbrella review.

#### Language

Limits will be set to only meta-analysis and systematic reviews in the English language due to the language capabilities of the study team.

### Exclusion criteria

Excluded studies will be based on the following criteria:

#### Population

Studies on non-human mammals only will be excluded.

#### Exposures

Reviews focusing on non-analgesic drugs or illegal drugs will not be included in this review, neither will studies examining neurodevelopmental disorders other than ASD or ADHD.

#### Study design

Articles that are not relevant to the review’s scope of prenatal use of analgesic drugs or children’s ASD or ADHD will be excluded. Types of articles which will be excluded are primary or original research, non-systematic reviews (e.g., narrative or scoping reviews), case studies or qualitative reviews, and reviews that draw on published opinion or theoretical studies as a primary source of evidence.

Two reviewers will assess studies against the inclusion and exclusion criteria.

### Search strategy

A search will be performed through major repositories of systematic reviews and meta-analyses, namely the following databases: Embase, Maternity and Infant Care, PsycINFO, PsycARTICLES, PubMed, and Cochrane Library. Boolean operators of “AND” and “OR” will be used for search terms and adapted for different databases. Search filters will be employed and presented sequentially for the databases with key terms searched for in the title or abstract fields. Relevant subject headings will also be used in addition to keywords (e.g., “Prenatal exposure drugs neurodevelopment” in the EMTREE thesaurus, or “Prenatal drugs effect ASD” in the MeSH thesaurus).

A list of example search terms is included in Additional File 3. Search periods will extend from the inception dates of the databases to the date of data extraction. The reference manager Zotero will be used to store records and identify duplicates.

In order to provide a comprehensive search, reference lists of selected reviews, reviews in-press (derived from scanning pre-print archives or discussion with field experts), will form part of the supplementary search strategy and recorded under “additional records.” These include contacting study authors, manual searches of gray literature (such as Open Grey, Virtual Health Library), and preprint platforms (such as arXiv.org, medRxiv.org, PsyArXiv.com, Open Science Framework [OSF] preprints).

### Screening and selection procedure

#### Data extraction

Data extraction and coding will be independently carried out by two researchers. All articles identified from the literature search will be independently screened by two researchers. First, titles and abstracts of articles will be screened based on the eligibility criteria as outlined above. Second, full texts will be examined in detail and screened for eligibility. Third, references of all considered articles will be searched manually to identify any relevant reports that may have been missed in the initial search strategy. Any disagreements will be resolved through discussion by the researchers to meet a consensus, if necessary. Information extracted from each study will include first author, year of publication, reported a protocol, objective(s), reported strategies to search literature, number of databases searched and date of last search, inclusion/exclusion criteria, population, main outcomes of interest, type of study designs included (e.g., observational studies), number of included studies, number of studies reporting data for meta-analyses, effect metric(s) reported (e.g., risk ratio), methods to assess study risk of bias, additional analyses, metabias assessment (e.g., publication bias across studies), funding source, and conflicts of interest. Extracted data will be stored in spreadsheets which will be used to determine eligibility for inclusion in the umbrella review.

Any discrepancies will be solved through discussion until consensus is achieved, with the assistance of the third researcher if needed. Studies with missing essential information such as participant data or search strategies will not be included in the final review. A PRISMA flow diagram showing the number of studies included and excluded at each stage of the study selection process will be provided [24] (Additional File 4). The umbrella review will undergo a full pilot process using a small sample of papers before a full review is initiated.

#### Quality assessment

A proposed quality assessment, the AMSTAR 2 (A MeaSurement Tool to Assess systematic Reviews-2) [26] will be used for this umbrella review (Additional File 2). This updated version of a critical appraisal tool was chosen for rapid quality assessments of systematic reviews in healthcare. The AMSTAR 2 tool consists of 16-items addressing search strategies, data extraction techniques, bias risk, appropriate methodology, and interpretation and discussion of results. Seven domains within the AMSTAR 2 are regarded as “critical” and flaws in these domains are deemed to affect the validity of the review (assessed by items 2, 4, 7, 9, 11, 13, 15). The AMSTAR 2 authors propose a scoring scheme which categorizes the confidence in the results of each review into “High,” “Moderate,” “Low,” and “Critically low.” We will use this scheme and reviews which we rate as “Critically low” (the study has more than one critical flaw) will be excluded from the data synthesis. Studies which we deem “Low” (one critical flaw) will be included, but their conclusions are given less weight.

The AMSTAR 2 not only allows for future replicability, but also provides reviewers with little epidemiological training with a standardized template, in order for a more in-depth appraisal of the literature. It consists of detailed questions. This tool is in line with advised guidelines on the assessment of systematic reviews based on identifying methodological features such as how well the research question is defined, use of a systematic search strategy, possible publication or funding bias, selective reporting, previous quality ratings, and presence of information synthesis and conclusion [27]. The AMSTAR 2 will be modified to suit the topic for this umbrella review through reducing the emphasis of randomized control trials in the quality assessment process.

#### Data synthesis

A narrative synthesis method is proposed for this umbrella review. Included studies will be tabulated with an overall summary. We will provide a narrative synthesis of the findings from each review, supported by a table showing the results of the critical appraisal based on included studies (as assessed by the modified AMSTAR 2 approach). The table will include a description of key features, findings, variations of research, and supplemented with graphics if relevant. A meta-analysis will not be performed. This method was primarily chosen due to the lack of firm conclusions on prenatal analgesic drug use and potential heterogeneity of data from multiple systematic reviews and meta-analyses. A narrative synthesis provides flexibility due to its qualitative rather than quantitative nature of the analysis. The purpose of this narrative synthesis is to examine and integrate ideas from multiple different reviews in order to provide a clear overview of the effects of analgesic drugs on neurodevelopmental diagnoses. Variations or discrepancies in findings will be explored by comparing methodological features between reviews (e.g., eligibility criteria, outcomes definitions).

#### Confidence in cumulative evidence

The strength of the body of evidence will be assessed through the Grading of Recommendations, Assessment, Development, and Evaluations (GRADE) certainty ratings. Using GRADE guidelines [28, 29], the researchers will examine the following domains for a decrease in certainty: risk of bias, imprecision of true effects, inconsistency of effects, indirectness of outcomes, and publication bias.

## Discussion

The purpose of this umbrella review is to elucidate the associations between prenatal use of analgesic drugs and children’s neurodevelopmental disorder diagnoses through the synthesis of relevant published research. Findings from this review will provide a clearer direction for clinical decisions made during pregnancy in relation to the use of analgesic drugs. A small sample of papers will be selected to conduct at the pilot as an initial stage of the umbrella review, including search strategies, inclusion/exclusion criteria, data extraction, and quality assessments. The results of the pilot will be used to refine data extraction in the full umbrella review or if necessary, modification of the scope of the review before proceeding with the full review.

Potential practical issues may present proposed search terms producing irrelevant results, in which the team will then redefine current search strategies. If key variables are missing from our data extraction plan, all amendments to the protocol will be documented and reported thoroughly. All amendments to the protocol will be documented in a separate summary sheet, which will be submitted in the final umbrella review.

A potential limitation of this review could involve difficulties in synthesizing information due to methodological differences of original studies leading to heterogeneous results in the selected reviews. Another limitation could arise from the emphasis of AMSTAR 2; due to its original focus of being a tool for randomized controlled clinical trials of interventions, several items in the AMSTAR 2 are not entirely relevant to this umbrella review, such as question 8 where the description of studies in adequate detail had to have “interventions” as an option to fulfill the criteria of “Yes” (Additional File 2). To mitigate this, the AMSTAR 2 will be modified to suit the topic of our umbrella review.

However, this also offers an opportunity to discuss these methodological differences and provide clear suggestions for future research. Using synthesized information on analgesic drugs and their association with neurodevelopmental outcomes, there are several strengths of this proposed review. A strength of the proposed review lies in the added value of synthesizing findings from previous reviews on the effects of prenatal use of analgesic medications on children’s long-term neurodevelopment. To our knowledge, no such review has been conducted on this topic as of yet. Findings could help refine clinical practices for the prenatal period and increase the quality of available information which future decisions in healthcare policy are based on.

### Protocol amendments

Any amendments made to the protocol will be documented throughout the review process and will also be discussed in the paper submitted for publication.

### Dissemination plans

The umbrella review will be written up and submitted for publication in a peer-reviewed journal. Findings will be presented at relevant conferences. We will work with the University of Edinburgh press and knowledge exchange offices on dissemination through press releases, targeted toward a wide audience, including policymakers.

## Supplementary information

**Additional file 1:.** Data extraction form.

**Additional file 2:.** AMSTAR 2.

**Additional file 3:.** Draft search.

**Additional file 4:.** Figure S1. PRISMA Flow Diagram.

**Additional file 5:.** PRISMA-P 2015 Checklist.

## Data Availability

Data available in a public (institutional, general, or subject-specific) repositories that issues datasets with DOIs (non-mandated deposition). Repositories include Embase, Maternity and Infant Care, PsycINFO, PsycARTICLES, PubMed, and Cochrane Library.

## Abbreviations

ASD: Autism spectrum disorder
ADHD: Attention deficit hyperactivity disorder
AMSTAR 2: A MeaSurement Tool to Assess systematic Reviews (2nd edition)
DSM-5: Diagnostic and Statistical Manual of Mental Disorders (5th edition)
FDA: Food and Drug Administration (USA)
PRISMA: Preferred Reporting Items for Systematic Reviews and Meta-Analyses
PROSPERO: International Prospective Register of Systematic Reviews
