# Supplementary material for: The association between analgesic drug use in pregnancy and neurodevelopmental disorders: protocol for an umbrella review
Source: Syst Rev. 2020 Sep 2;9:202. doi: 10.1186/s13643-020-01465-9 (PMC7469356; doi:10.1186/s13643-020-01465-9)
Supplement: Supplementary file 3 — Additional file 3:. Draft search. [file 13643_2020_1465_MOESM3_ESM.docx]

Example search strategy:

1. Open PubMED database.
2. Authors to search for systematic reviews on aspirin taken during pregnancy and how it affects neurodevelopment.
3. Search terms will include: (“fetus” OR “child” OR “mothers” OR “pregnancy” OR “antenatal”) AND (“analgesics” OR “painkillers” OR “drugs” OR) AND (“medication” OR “aspirin”) AND (“neurodevelopment” OR “child development” OR “autism” OR “attention-deficit hyperactivity”) AND (“meta-analysis” OR “systematic review” OR “quantitative review”).

Filters applied: journal articles or book chapters only; publication dates from database inception to [date of search], human species only, English language only

**Examples of search terms.**

| # | Category | Search Terms | |
| --- | --- | --- | --- |
|  |  |  |  |
| 1 | Population | 1 | Fetus* OR |
|  |  | 2 | Child OR |
|  |  | 3 | Offspring |
|  |  | 4 | Mother* OR |
|  |  | 5 | Pregnan* OR |
|  |  | 6 | Prenatal OR |
|  |  | 7 | Maternal OR |
|  |  | 8 | Antenatal* |
| 2 | Exposures | 1 | Analgesics* OR |
|  |  | 2 | Painkillers* OR |
|  |  | 3 | Drugs OR |
|  |  | 4 | Medication |
|  |  | 5 | Aspirin OR |
|  |  | 6 | Acetaminophen OR |
|  |  | 7 | Ibuprofen OR |
|  |  | 8 | Naproxen OR |
| 3 | Outcomes | 1 | Neurodevelopment* OR |
|  |  | 2 | Child Development* OR |
|  |  | 3 | “Autism” OR |
|  |  | 4. | Asperger OR |
|  |  | 5 | “Attention-Deficit Hyperactivity” OR |
|  |  | 5 | Inattention OR |
|  |  | 6 | Hyperactivity OR |
|  |  | 7 | Impulsivity OR |
|  |  | 8 | Hyperkinetic |
| 4 | Review Design | 1 | “Meta-analysis” OR |
|  |  | 2 | “Systematic Review” OR |
|  |  | 3 | “Quantitative review” OR |
|  |  | 4 | Synthesis |
