# Supplementary material for: The association between analgesic drug use in pregnancy and neurodevelopmental disorders: protocol for an umbrella review
Source: Syst Rev. 2020 Sep 2;9:202. doi: 10.1186/s13643-020-01465-9 (PMC7469356; doi:10.1186/s13643-020-01465-9)
Supplement: Supplementary file 4 — Additional file 4:. Figure S1. PRISMA Flow Diagram. [file 13643_2020_1465_MOESM4_ESM.docx]

**Figure 1. PRISMA Flow Diagram**

Additional records identified through other sources

(n = )

Records identified through database searching
(k = )

## Included

## Eligibility

## Screening

## Identification

Studies included in quantitative synthesis (meta-analysis)
(n = )

Studies included in qualitative synthesis
(n = )

Full-text articles assessed for eligibility
(n = )

Full-text articles excluded, with reasons
(n = )

Records excluded with additional limits set
(n = )

Records screened
(n = )

Records after duplicates removed
(n = )
